# A comprehensive analysis of auditory event‐related potentials and network oscillations in an NMDA receptor antagonist mouse model using a novel wireless recording technology

**DOI:** 10.14814/phy2.13782

**Published:** 2018-08-28

**Authors:** Niklas Schuelert, Cornelia Dorner‐Ciossek, Michael Brendel, Holger Rosenbrock

**Affiliations:** ^1^ CNS Diseases Research Germany Boehringer Ingelheim Pharma GmbH & Co. KG Biberach an der Riss Germany; ^2^ Biostatistics and Data Sciences Boehringer Ingelheim Pharma GmbH & Co. KG Biberach an der Riss Germany

**Keywords:** Animal model, event‐related potentials, gamma oscillation, NMDA receptor antagonist, schizophrenia, translational biomarker

## Abstract

There is growing evidence that impaired sensory processing significantly contributes to cognitive deficits found in schizophrenia. Electroencephalography (EEG) has become an important preclinical and clinical technique to investigate the underlying mechanisms of neurophysiological dysfunctions in psychiatric disorders. Patients with schizophrenia show marked deficits in auditory event‐related potentials (ERP), the detection of deviant auditory stimuli (mismatch negativity, MMN), the generation and synchronization of 40 Hz gamma oscillations in response to steady‐state auditory stimulation (ASSR) and reduced auditory‐evoked oscillation in the gamma range. Due to a novel data‐logging technology (Neurologger, TSE Systems), it is now possible to record wireless EEG data in awake, free‐moving small rodents without any restrictions due to size of the device or attached cables. Recently, a new version of the Neurologger was released with improved performance to record time‐locked event‐related EEG signals. In this study, we were able to show in mice that pharmacological intervention with the NMDA receptor antagonists Ketamine and MK‐801 can impair a comprehensive selection of EEG/ERP readouts (ERP N1 amplitude, 40 Hz ASSR, basal and evoked gamma oscillation, MMN) and therefore mimic the EEG deficits observed in patients with schizophrenia. Our data support the translational value of NMDA receptor antagonists as a model for preclinical evaluation of sensory processing deficits relevant to schizophrenia. Further, the new Neurologger system is a suitable device for wireless recording of clinically relevant EEG biomarkers in freely moving mice and a robust translational tool to investigate novel therapeutic approaches regarding sensory processing deficits related to psychiatric disorders such as schizophrenia.

## Introduction

Schizophrenia is a severe psychiatric disorder that affects about 1% of the population worldwide. At present, there are no approved treatments that specifically target the neurocognitive impairment in schizophrenia. Therefore, there is the pressing need to understand the neural mechanisms underlying these deficits to improve treatment options. Recent research has shown that the integration and processing of sensory information is altered in schizophrenia.

The NMDA receptor is a cation channel that produces excitatory postsynaptic potentials when activated and is important for learning and synaptic plasticity (Wong et al. 1986). NMDA receptor antagonists such as ketamine, MK‐801, or phencyclidine have been shown to induce an array of transient symptoms in animals and humans that mimic symptoms of patients with schizophrenia (Newcomer et al. 1999; Olney et al. 1999; Saunders et al. 2012a) and they were also shown to exacerbate symptoms in patients (Lahti et al. 1995). In addition, NMDA receptor antagonists induce EEG deficits in animals and humans similar to deficits observed in patients with schizophrenia (Todd et al. 2006; Ehrlichman et al. 2008; Javitt and Sweet 2015; Harms 2016). Accordingly, deficits in NMDA receptor function are thought to contribute to the neurobiology of schizophrenia (Gonzalez‐Burgos and Lewis 2012; Dawson et al. 2014). Most of the biomarkers validated in this study are closely linked to NMDA receptor function.

The processing of sensory inputs can be analyzed by using EEG to record auditory event‐related potentials (ERP) at the brain level. To measure ERPs, a large number of time‐locked experimental trials are averaged and the potential, composed of successive positive and negative deflections is detectable. Therefore, ERPs provide a functional measure of brain activity that occurs time‐locked to an external event, reflecting successive stages of information processing. These deflections can vary in amplitude and latency, depending on the neurological condition. For example, ERP recorded in patients with schizophrenia present smaller amplitudes as compared to healthy subjects. EEG recordings analogous to those in humans can be recorded from a variety of rodent species. In mice, the characteristic positive and negative deflections of the EEG recording occur at approximately 40% of the latency of equivalent human components (Siegel et al. 2003; Connolly et al. 2004). Therefore, the P20, N40, P80 and P120 represent ERP deflections in mice analogous to the P50, N100, P200 and P300, respectively, in humans. Due to the obvious limitations of animal models for schizophrenia, in this study we focused on the validation of electrophysiological biomarkers that allow translational measures both in preclinical models and in clinical studies.

The best established measurement for the study of auditory sensory dysfunction in schizophrenia is the auditory mismatch negativity (MMN) paradigm. The MMN is a widely studied ERP component that reflects preattentive processing of the relationship between successive auditory stimuli. A physiological NMDA receptor signaling is crucial for a neuronal network to enable MMN (Tikhonravov et al. 2008; Harms 2016; Lee et al. 2017). A sequence of repetitive stimuli (standards) is interrupted by a physically different (deviant) stimulus. This deviant stimulus elicits a bigger ERP response and the difference to the standard ERP is defined as MMN (Light and Swerdlow 2015; Harms 2016). In addition to amplitude measures of ERPs, by analyzing the auditory‐evoked oscillations, the frequency components can be extracted. Cortical gamma oscillations are linked with a variety of cognitive processes like perception, working memory and attention (Bertrand and Tallon‐Baudry 2000; Herrmann et al. 2004). Significant alterations of the electrical activity in the gamma band have been documented in patients with schizophrenia (Minzenberg et al. 2010; Spencer 2011; Uhlhaas and Singer 2013) and in different animal models (Vohs et al. 2010). Spontaneous gamma oscillation has been shown to be elevated in schizophrenia patients (Baldeweg et al. 1998; Behrendt 2003; Spencer 2011) while the evoked power responses that are phase‐locked to sensory stimulus appear to be diminished (Kwon et al. 1999; Bertrand and Tallon‐Baudry 2000; Leicht et al. 2010; Hudson et al. 2016). Since there is a strong correlation between impaired gamma oscillation and cognitive deficits in schizophrenia, gamma band deficits appear to be a promising biomarker for diagnostic purposes as well as for drug testing of novel antipsychotic compounds. In recent studies of evoked oscillations in schizophrenia, the auditory steady‐state response (ASSR) paradigm was used to examine the ability of the cortex to maintain an entrainment of 40 Hz oscillation. There is a significant reduction in the power as well as in the phase‐lock coherence in 40 Hz ASSR in schizophrenia patients (Brenner et al. 2009; Thune et al. 2016). In addition, evoked oscillations analysis allows the measurement of intertrial coherence (ITC), representing the capacity of the sensory network to stereotypically process a repeated input. Gamma oscillation is thought to reflect the interplay between cortical pyramidal neurons and parvalbumin‐expressing interneurons (Moghaddam et al. 1997; Razoux et al. 2007). Since a reduction of ASSR power has been observed in Schizophrenia, it is suggested that this is due to impairment in the pyramidal‐parvalbumin interneuron circuitry (Gonzalez‐Burgos and Lewis 2012). In rodents, impairments of ASSR power and phase‐lock coherence can also be induced by NMDA receptor antagonists (Sivarao et al. 2013, 2016) which could be explained by disinhibition of pyramidal neurons and thus disturbed excitatory/inhibitory (E/I) balance.

NMDA receptor antagonists like MK‐801 or ketamine are used as pharmacological tools to induce behaviors that are related to positive, cognitive and negative symptoms in schizophrenia (Kocsis et al. 2013; Neill et al. 2014). In rodents, acute injections of ketamine produce neurobiological features very similar to this psychiatric disorder: hyper‐dopaminergic state in basal ganglia, memory impairment, social interaction deficits, prepulse inhibition deficits, and increased cortical gamma oscillation (Phillips et al. 2012). Moreover, ketamine induces modifications of ERP and evoked oscillation features in mice that are very similar to those observed in patients with schizophrenia: reduction of the ERP amplitude and evoked gamma power (Featherstone et al. 2012; Saunders et al. 2012a,b). Thus, NMDA receptor antagonists such as ketamine seem to be a perfect pharmacological tool to compare the effect of drug candidates on the sensory processing and E/I‐balance.

In this study, we used for the first time a wireless recording system for the recording of the most relevant event‐related EEG readouts applied in schizophrenia research and diagnosti**c.** A challenge in preclinical EEG research is the fact that use of custom‐made recording devices as well as self‐programmed routines for analysis makes a transparent comparison and reproduction of study designs very difficult.. To improve the reproducibility and transparency of our data analysis, we used exclusively commercially available analysis software which is used broadly in the clinical setting and by providing a detailed description of protocols and analysis improving transparency and enable reproducibility. We also showed that all relevant readouts can be reliably recorded using surface electrodes improving the translational value. We tested two different NMDA receptor antagonists for all readouts to validate the observed effects. Ketamine and MK‐801 are both receptor antagonists and noncompetitive inhibitors, but MK801 has a much lower reversibility than ketamine. Additionally, MK801 has 160 times the affinity of ketamine, necessitating higher ketamine doses for similar drug effect. In addition, MK801 has a longer CNS exposure which is beneficial for a stable drug exposure during the test protocols. Because EEG biomarkers as ERP, MMN, or ASSR can be elicited under conditions in which attention to stimuli is not required, these measures seem to be an ideal translational biomarker to test pharmacological compounds on rodent models that target certain aspects of human psychiatric disorders by assessing the integrity of relevant neuronal circuits.

## Material and Methods

### Compounds and pharmacological treatment

The NMDA receptor antagonists Ketamine (S‐ketamine hydrochloride) and MK‐801 (hydrogen maleate) were purchased from Sigma. Drug solutions were prepared 24 h before injection. To prepare the solutions, appropriate amounts of drug salt were weighed, dissolved in vehicle consisting of 0.9% (weight/volume) NaCl solution, and stored at 4°C until use. Drug solutions or vehicle was administered subcutaneously (s.c.) 10 min before start of auditory stimulation protocols; the following doses were used: Ketamine 10 mg/kg and MK‐801 at 0.03 mg/kg, 0.1 mg/kg or 0.3 mg/kg. Only for the MMN protocol, a highest dose of 0.2 mg/kg MK‐801 was applied since the 0.3 mg dose resulted in a complete absence of either standard or deviant potentials in a previous test. Twelve animals received MK‐801 in three different concentrations. Additionally, 12 animals received vehicle in parallel to the MK‐801‐treated group (not shown in the study). This vehicle cohort was used for our internal validation, to ensure that the different readouts are consistent over the time of the study and that there are no time‐related shifts. This vehicle cohort was used subsequently for the Ketamine study. At the beginning of the study, all animals in both groups received vehicle and all EEG readouts were measured to get a recording baseline. After 3 days, the MK‐801 treatment group received the lowest dose of MK‐801 (0.03 mg/kg) and the vehicle control group the vehicle. After 3 days of washout, the next higher doses were administered and the protocols repeated. At the end, all animals in both groups received another dose of vehicle to obtain data after washout. After completion of the MK‐801 study, in the vehicle control group all stimulation protocols were tested with application of 10 mg/kg of Ketamine and a washout recording with vehicle after 3 days. Injections and recordings were performed twice a week, to allow a minimum washout period of 3 days between injections. Determination of drug levels in pilot PK experiments revealed that both drugs are completely eliminated from plasma and brain after 24 h.

The following sequence of treatments was applied to animals in the two treated groups.

#### MK‐801‐treated group

Vehicle at baseline

1. MK‐801 0.03 mg
2. MK‐801 0.1 mg
3. MK‐801 0.3 mg/MK‐801 0.2 mg (only for MMN protocol)
4. Vehicle after final washout.

#### Vehicle‐treated control group

1. Vehicle
2. Vehicle
3. Vehicle
4. Vehicle
5. Vehicle after final washout.

#### Ketamine treatment of vehicle‐treated control group

1. Vehicle at baseline
2. Ketamine 10 mg
3. Vehicle after final washout.

### Animals

Animals used were C57Bl/6 mice, weight 25–28 g upon arrival, provided by Janvier (France). Animals were first housed in group cages (maximum five animals per cage), with free access to food and water. The animal facility was maintained under artificial lighting (12 h; light on at 6 am, light off at 6 pm), with both controlled ambient temperature and relative humidity. A 1‐week habituation period was allowed before surgery. EEG recordings of animals took place during light phase. After surgery, animals were kept single‐housed for the rest of the study. All experimental procedures were authorized by the Local Animal Care and Use Committee in accordance with local animal care guidelines, AAALAC regulations and the USDA Animal Welfare Act.

### Electrode implantation

For electrode implantation, animals were anaesthetized using isoflurane (2–3% in oxygen) and placed in a stereotaxic frame with a controlled heating blanket. Under sterile conditions a longitudinal incision was made and the skin flaps were retracted with clamps. The surface of the scull was cleaned with hydogen peroxide solution (5%) and dried off with air puffer. Three small holes were drilled into the scull without damaging the dura mater. Coordinates for recording from the auditory cortex were −2.7 mm posterior, 4 mm lateral. The hole for the reference electrode was positioned above the cerebellum at −1.5 mm posterior to lambda. Then, three gold‐plated steel screws each with a connector cable and a metal pin were implanted into the cranium superficially of the dura mater. A socket with the connector pins to attach the Neurologger was implanted and fixed with dental cement. At the end of the surgery, animals received an injection of Metacam (0.01 mg/kg i.p.) and Baitril Antibiotics s.c. over 5 days. After surgery, animals were maintained in individual cages, and left to recover for 1 week.

### Animal selection

A preliminary EEG recording was performed 1 week after surgery. An ERP protocol was used to determine quality and reliability of recordings in baseline conditions after vehicle administration (see below for condition description). To ensure quality of recordings and proper placement of electrodes, only animals with minimal (100 *μ*V) P1N1 amplitude (see below for definition) were included in the study. Using this criterion, 24 animals were selected for the study. Animals were randomly assigned to two treatment groups (MK‐801 or Ketamine).

### Setup specifications

**Figure 1:**
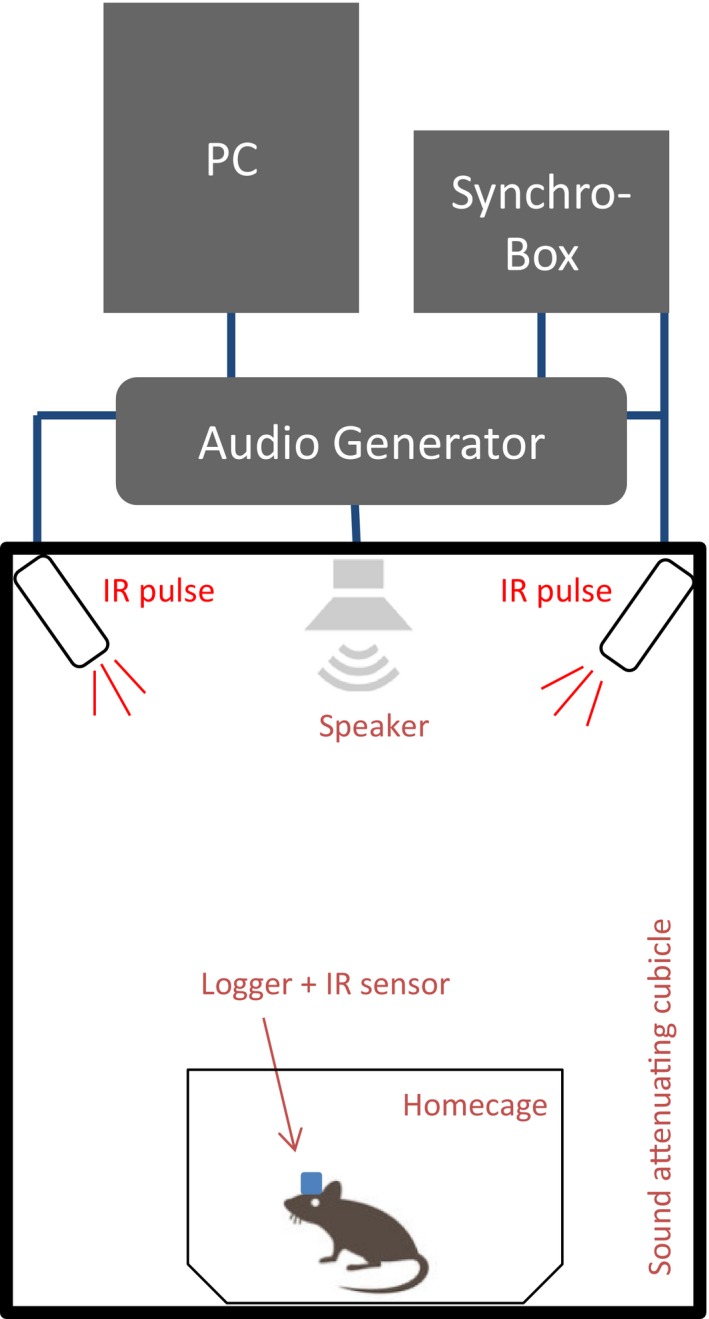
Experimental setup for auditory stimulation and EEG recording in a noise‐attenuated cubicle. IR (infrared) diodes that emit an infrared pulse time‐locked to the auditory stimulus are placed on the ceiling of the cubicle. The loudspeaker for presentation of auditory stimulation protocols is installed at the ceiling of the cubicle above the testing‐cage of the animal.

Auditory stimuli were generated with an audio generator (MED Associates Inc.) and presented via a house speaker system in each of the sound‐attenuated boxes (MED Associates Inc.). Each box was equipped with a halogen LED house light (Fig. 1).

Two Infrared LED lights (TSE systems) were installed in a 45° angle on the ceiling of the cubicle to cover the entire interior. The infrared diodes were connected to a synchro‐box (TSE systems), which was connected to the audio generator. Using MED‐specific software, the auditory stimulation protocols were programmed and a TTL signal was sent simultaneously with each tone to activate the LED infrared lights. An infrared sensor on the Neurologger recorded the infrared trigger signals and trigger signals were saved in an individual trigger channel.

### Auditory stimulation protocols

#### Event‐related potential (ERP) recordings and evoked/basal oscillation

**Figure 2:**
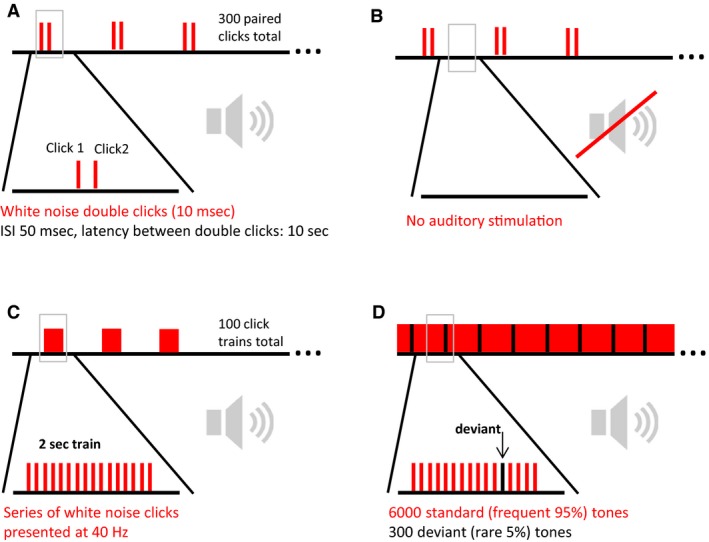
Different auditory stimulation protocols for (A) auditory event‐related potentials (ERP) and evoked oscillation recording (B) basal oscillation without auditory stimulation (C) Auditory steady‐state response (ASSR) recording and (D) mismatch negativity (MMN) recording.

ERP session consisted of 300 repetitions of paired clicks. A click consisted of a 10 msec white noise sound emitted at 85 dB with a 1 msec rise and fall (Fig. 2A). Sound pressure was measured by placing a sound pressure meter (MED Associates) on the bottom of the cage. Paired clicks were separated by 0.5 sec. Pairs were separated by 10 sec. A complete ERP session lasted approximately 45 min. This protocol was also used to measure evoked gamma and theta oscillation. The EEG segments in the phase between a paired click were used to analyze the basal gamma and theta power (Fig. 2B).

#### 40 Hz auditory steady‐state response (ASSR) recordings

An ASSR session consisted of periodic train of single white noise clicks in a frequency of 40 Hz (Fig. 2C). Each train lasted 2 sec with an interval of 30 sec in between click trains. A total of 100 trains were presented. The intensity of the 40 Hz click train was adjusted to be 85 ± 1.0 dB.

#### Mismatch negativity (MMN)

An auditory oddball paradigm was used to induce MMN. Mice were presented with a series of standard tones (9 or 12 kHz) in which a novel tone (9 or 12 kHz) was intermittently presented (Fig. 2D). The ratio of standard to deviant tone was 19–1 (5%) with deviant tones being presented every 20th trial. A total of 12,000 standard and 600 deviant tones were presented per trial. The frequency of the standard and the novel tones were switched after 50% of presented stimuli, such that all animals received the 9 and 12 kHz tone as both standard and deviant with an intensity of 85 dB, described previously as a flip‐flop design (Harms et al. 2016). Tone length was 50 ms and they were separated by a 0.5 sec inter‐stimulus‐interval (ISI). The MMN protocol lasted around 100 min. MMN area under the curve was analyzed 25 msec before and 25 msec after the peak amplitude as described (Beauchemin 2005).

### Recording procedure

Neurologgers were attached to the implanted connector pins 30 min before start of stimulation protocols. After attachment of the Neurologger, animals were individually placed in an open home cage in sound‐attenuated recording boxes (MED Associates Inc.). The bottom of the cage was covered with a cotton tissue for rodent cages. To minimize the effects of stress, animals were given 30 min to acclimate to the apparatus prior to each recording session. All 24 animals were always tested on the same day.

On test day 1, a baseline recording was started 10 min after vehicle injection for all 24 animals. On test day 2, the first dose of NMDA antagonist (either MK‐801 or ketamine) was administered and recording was started 10 min after injection. After a recovery period of at least 3 days, the next dose of MK‐801 was applied. Only 1 protocol and 1 dose of compound were tested per test day.

### Data analysis

To retrieve the data from the Neurologger, a TSE readout station was used and data were transformed into and EDF file via TSE internal software. Two recorded channels and one trigger channel were analyzed per animal (two channels for each auditory cortex and a trigger channel to identify the time points of tone presentations. The sampling rate of recording channels and trigger channels was set to 1000 Hz. The quality of EEG recordings was carefully checked and segments with artifacts were removed. Data were analyzed using the Analyzer2 software package (BrainProducts GmbH, Munich, Germany).

#### Analysis of ERPs

ERPs were recorded with a modified protocol similar used in previous preclinical and clinical studies (Connolly et al. 2004; Rissling et al. 2010; Javitt and Sweet 2015). To analyze ERPs, the file was segmented into 300 segments with a bin length of 1200 msec (200 msec prestimulus and 1 sec poststimulus). Segments were normalized to baseline and the average was calculated. Positive and negative deflections of the ERP were defined according to established literature (Maxwell et al. 2006; Rissling and Light 2010; Javitt and Sweet 2015). The N1 deflection was defined as the maximum negative deflection located between 30 and 50 msec after a click. Sensory gating of the N1 amplitude between paired click stimuli was measured by calculating the ratio between the N1 amplitude of the response evoked by the click 1 and the N1 amplitude of the response evoked by the click 2. This index gives an indication of the capability of the sensory system to filter out repeated stimuli which is reflected in the reduced amplitude of the redundant stimulus.

#### Analysis evoked and basal oscillation

Evoked oscillations were recorded with a modified protocol similar used in previous preclinical and clinical studies (Saunders et al. 2012a; Javitt and Sweet 2015; Sullivan et al. 2015b; Hudson et al. 2016). Evoked oscillation was analyzed and quantified with the Analyzer2 software package performing a continuous wavelet transform with complex Morlet function. The basal power was defined as the power of a specific frequency band outside of the auditory‐evoked response. It is quantified using a 400 msec bin 3 sec before click presentations. The evoked gamma power in the frequency band 35–80 Hz is defined as the total power (evoked plus basal power) subtracting the basal power. The gamma frequency band was calculated from the 50 msec time frame surrounding the P1 peak (25 msec prepeak and 25 msec postpeak). The evoked theta power in the frequency band 4–12 Hz is defined as the total power of the frequency band measured during a 200 msec time frame surrounding the P1 peak (20 msec prepeak and 180 msec postpeak) and subtracted the basal power in this frequency band. A continuous wavelet transformation was conducted using BrainVision Analyzer2 software (v 2.1.1, Brain Products GmbH). A complex Morlet wavelet was used to decompose the EEG signals for in the range of gamma (35–80 Hz) oscillation. The step between successive frequencies was set at 1 Hz and a logarithmic subdivision of the selected frequency layers was selected. The Morlet parameter was set at *c* = 5. The data were baseline‐corrected to the basal oscillation 3 sec before a stimulus event. The spectral evoked as well as basal power was computed as real values (*μ*V^2^). Output numbers are real numbers and contain information about the spectral power for each frequency layer and time point. Phase information is not considered. By using the P1 peak as reference point, individual variation in the location of the narrow gamma band was considered. Quantifications were normalized to the average power of the same frequency band over the whole recording.

#### Analysis ASSR

For recording of auditory steady‐state responses, a modified protocol of previously published studies was used (Brenner et al. 2009; Vohs et al. 2010; Spencer 2011; Sivarao et al. 2013, 2016; Sivarao 2015; Thune et al. 2016). For analyzing the auditory steady‐state response (ASSR), data were filtered using a second‐order Butterworth IIR zero phase‐shift band‐pass with a higher and lower cut off adjusted to 5 Hz above and below the stimulus frequency (35 and 45 Hz for the 40 Hz stimulus). Data were segmented into 2.8 sec epochs with 400 msec pretrial period, 2 sec trial period and 400 msec posttrial period. A Morlet wavelet analysis was performed for the 40 Hz frequency layer to measure mean power and intertrial coherence (ITC) during the trial period. ASSR power was expressed as the accumulated power of the 40 Hz layer during the stimulation phase of 2 sec. For the ITC wavelet, coefficients with complex numbers were computed and the phase‐locking factor was determined by averaging the normalized phase synchronization across trials for every time point and frequency. The event‐related changes in power relative to a prestimulus baseline reflect the amplitude of stimulus evoked gamma, while the ITC indicates phase consistency across trials independent of signal amplitude and ranges between 0 (nonphase‐locked, random activity across trials) and 1 (activity that is fully locked in phase across individual trials). We used evoked power and ITC, respectively, because these parameters provide information about the temporal dynamics of ASSR. Decreases of AERO and/or intertrial coherence reflect reduced neural responses to auditory steady‐state stimulation.

#### Analysis MMN

MMN was defined according to previously published preclinical and clinical studies (Rissling and Light 2010; Light and Swerdlow 2015; Harms 2016; Featherstone et al. 2018). It was calculated by subtracting the averaged response to the set of standard stimuli from the averaged response to the deviant stimuli. To ensure that the resulting ERP was derived from an equal number of standard and novel tones, only the last standard tone prior to the deviant tone was used. The difference was calculated by subtracting the potential of the standard ERP from the deviant ERP. In the resulting negative deflection, the MMN component was obtained using the area under the curve contained within a 50 msec time window (±25 msec from the peak amplitude) in which the peak MMN had previously been identified by the peak detection function in Analyzer2 as described in a clinical study (Beauchemin 2005). By centering this defined window around the peak amplitude accounts for the greatest difference between the standard and the deviant stimulus.

### Statistics

All statistical analyses were conducted separately for each compound (Ketamine, MK‐801) and parameter. The descriptive statistics were calculated and the data were visualized and assessed (not shown in this work). Assuming that the considered data follow normal distributions, a mixed model was fitted to the data. The model comprises of a random intercept for each animal and the applied treatment as a fixed factor. The following relevant comparisons were estimated together two‐sided confidence intervals (confidence level *γ* = 0.95).

#### MK‐801‐treated group

1. MK‐801 0.03 mg – Vehicle at baseline
2. MK‐801 0.1 mg – Vehicle at baseline
3. MK‐801 0.3 mg – Vehicle at baseline/MK‐801 0.2 mg – only for MMN ‐Vehicle at baseline
4. Vehicle after final washout – Vehicle at baseline.

#### Ketamine‐treated group

1. Ketamine 10 mg – Vehicle at baseline
2. Vehicle after final washout – Vehicle at baseline.

In order to account for multiple testing, the statistical significance was assessed by comparing the *P*‐values with a Bonferroni corrected significance level. The applied Bonferroni correction does not account for the number of investigated compounds (Ketamine/MK‐801) and the number of auditory stimulation protocols (ERP, ASSR, MMN), as these factors are regarded as independent scientific questions. The applied Bonferroni correction accounts for the number of tested dosages (Ketamine: 1, MK‐801: 3) and the number of considered parameters (ERP: 6, ASSR: 2, MMN: 1). This procedure provides the following Bonferroni corrected significance levels α* = 0.05/6 ≈ 0.008333 (ERP), α* = 0.05/2 = 0.025 (ASSR), α* = 0.05/1 = 0.05 (MMN) for Ketamine and α* = 0.05/(3∙6) ≈0.002777 (ERP), α* = 0.05/(3∙2) ≈ 0.008333 (ASSR), α* = 0.05/(3∙1) ≈ 0.016666 (MMN) for MK‐801.

The comparison of vehicle after final washout and baseline is not considered in the Bonferroni correction.

The statistical evaluation was prepared using the software package SAS Version 9.4 (SAS Institute Inc., Cary, North Carolina, USA).

The visual analysis of the data in the vehicle‐treated control group showed that the level of the measurements is constant during the experiment (data not shown). Even though the control group data are part of the analysis model, only the comparisons in the MK‐801 treated group are shown. The comparison between the MK‐801‐treated group and the vehicle control group essentially provides the same overall picture, but the shown difference within the MK‐801‐treated group allows for the comparison with the results obtained from the Ketamine study. The different protocols and the tested compounds are considered as independent scientific questions; therefore, these factors were not considered in the correction for multiple testing. Only the tested dosages and the measured parameter within a protocol are considered in the applied Bonferroni correction. The comparison vehicle after final washout and baseline is not subject to a correction for multiple testing, as this comparison is used as a check, if the mice returned at the end of the experiment to their original level or if the data obtained after final Washout indicate on a change in level of the measurement during the course of the studies.

## Results

### ERP

#### N1 amplitude

**Figure 3:**
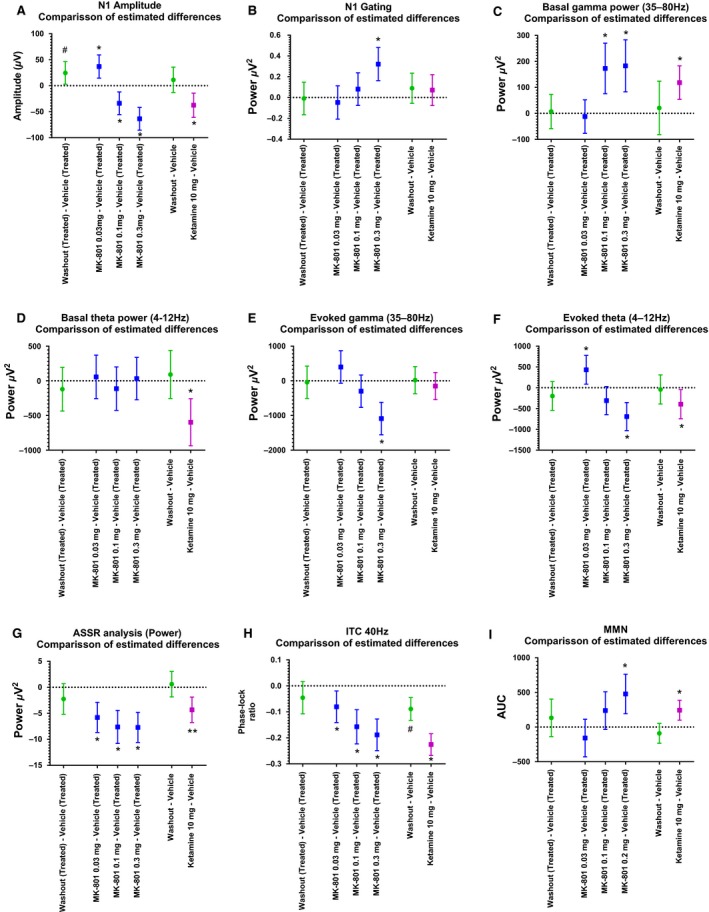
Estimated differences and confidence limits at the significance level (confidence level *γ* = 0.95) on relevant comparisons based on mixed model for the different (A) N1 amplitude; (B) N1 gating; (C) basal gamma; (D) basal theta; (E) evoked gamma; (F) evoked theta; (G) ASSR power (H) ASSR ITC; (I) MMN. *Statistically significant after correction for multiple testing (applies only for the comparisons compound vs. vehicle). ^#^Statistically significant at 5% level (applies only for the comparisons washout vs. vehicle).

**Figure 4:**
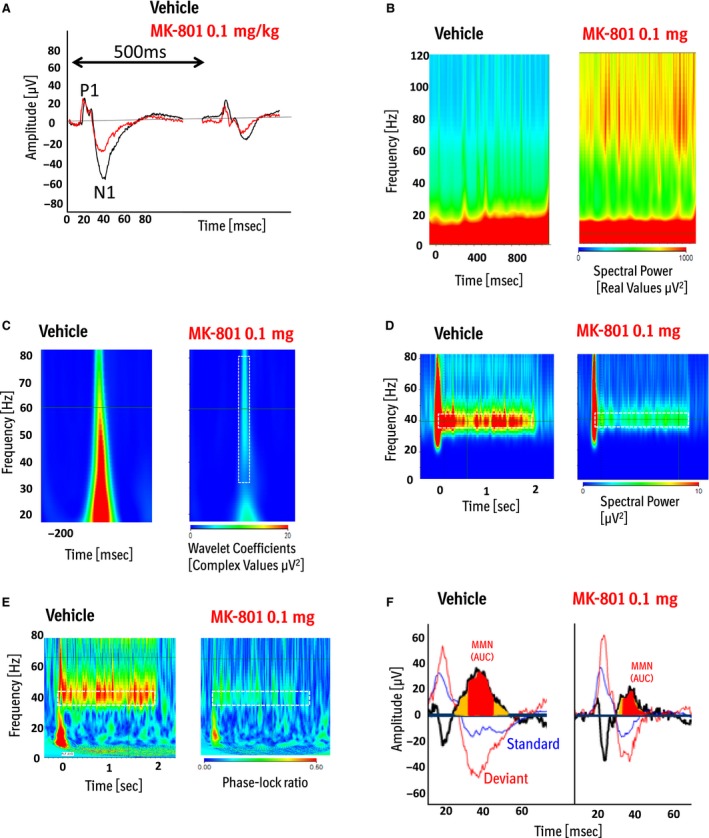
Exemplary readouts for one individual animal before and after application of MK‐801 for (A) N1 amplitude; (B) basal gamma power; (C) evoked gamma power; (D) auditory steady‐state power; (E) auditory steady‐state intertrial coherence (ITC) and (F) mismatch negativity MMN (area under the curve).

The comparison of vehicle after final washout and baseline revealed a significant increase of the N1 amplitude in the MK‐801‐treated group (Fig. 3A and 4A). The concentration of 0.03 mg MK‐801 induced a significant increase in the N1 amplitude (Estimated difference: 36.9; confidence limits: 14.6–59.3; *P* = 0.0015) compared to vehicle at baseline and the concentrations of 0.1 mg (Estimated difference: −33.74; confidence limits: −55.51 to −11.96; *P* = 0.002773) and 0.3 mg MK‐801(Estimated difference: −63.59; confidence limits: −85.37 to −41.81; *P* < 0.0001) induced a significant decrease in N1 amplitude. Ketamine 10 mg induced a significant decrease in N1 amplitude (Estimated difference: −37.48; confidence limits: −60.7 to −14.27; *P* <= 0.0025) compared to vehicle at baseline. In the MK‐801‐treated group, the N1 amplitude was significantly increased after washout compared to vehicle at baseline (Estimated difference: 24.61; confidence limits: 2.84–46.39; *P* < 0.05). Vehicle at baseline compared to after the final washout was not significant in the Ketamine‐treated group (Estimated difference: 11.3; confidence limits: −13.07–35.69; *P* = 0.35).

#### Gating of N1

Only the highest dose 0.3 mg MK‐801 significantly reduced the gating ratio (Estimated difference: 0.3214; confidence limits: 0.1616–0.4811; *P* = 0.0002). Ketamine 10 mg had no significant effect on N1 gating (Estimated difference: 0.07; confidence limits: −0.076–0.21; *P* = 0.33). Vehicle at baseline was not significant to vehicle after final washout in neither the MK‐801 group nor the ketamine group (*P* > 0.05) (Fig. 3B).

### Event‐related oscillations

#### Basal gamma power

The concentration of 0.03 mg MK‐801 had no effect on basal gamma power compared to vehicle at baseline (Estimated difference: −12.32; confidence limits: −115.1–90.45; *P* = 0.81). The concentrations of 0.1 mg (Estimated difference: 172.83; confidence limits: 75.54–270.11; *P* = 0.0007) and 0.3 mg MK‐801 (Estimated difference: 182.86; confidence limits: 82.98–282.75; *P* < 0.0005) induced a significant increase in basal gamma power. Ketamine 10 mg also induced a significant increase in gamma power (Estimated difference: 118.31; confidence limits: 53.84–182.78; *P* = 0.0010). No significant differences were detected comparing vehicle at baseline and after final washout neither in the MK‐801 group nor in the Ketamine group (*P* > 0.05) (Fig. 3C and 4B).

#### Basal theta power

None of the three doses of MK‐801 had a significant effect on basal theta power. However, Ketamine induced a significant increase in basal theta (Estimated difference: (−597.76; confidence limits: −936.93 to −258.59; *P* = 0.0014). No significant differences were detected comparing the vehicle at baseline and after the final washout neither in the MK‐801 group nor in the ketamine group (*P* > 0.05) (Fig. 3C).

#### Evoked gamma power

Only the highest dose of MK‐801 significantly reduced evoked gamma power compared to vehicle at baseline (Estimated difference: −598.96; confidence limits: −962.39 to −235.53; *P* = 0.0016). Ketamine had no significant effect on evoked gamma (Estimated difference: −19.42; confidence limits: −288.68–249.83; *P* = 0.88). Vehicle at baseline was not significant to vehicle after the final washout in neither the MK‐801 group nor the ketamine group (*P* > 0.05) (Fig. 3D and 4C).

#### Evoked theta power

The 0.03 mg dose MK‐801 induced an increase in evoked theta compared to vehicle at baseline (Estimated difference: 432.24; confidence limits: 85.61–778.88; *P* = 0.0154). The dose of 0.1 mg MK‐801 induced a decrease (Estimated difference: −309.61; confidence limits: −646.47–27.24; *P* = 0.07) while the highest 0.3 mg dose MK‐801 induced a significant decrease in evoke theta (Estimated difference: −692.42; confidence limits: −1029.28–−355.57; *P* < 0.0001). Ketamine induced a decrease in evoked theta (Estimated difference: −395.0; confidence limits: −745.0 to −45.0; *P* = 0.03). Vehicle at baseline was not significant to vehicle after the final washout in neither the MK‐801 group nor the ketamine group (*P* > 0.05) (Fig. 3E).

#### 40 Hz ASSR power

All three doses of MK‐801 induced a significant reduction of 40 Hz ASSR power **(**0.03 mg: estimated difference: −5.78; confidence limits: −8.68 to −2.88; *P* = 0.0002; 0.1 mg: estimated difference: −7.62; confidence limits: −10.79 to −4.44; *P* < 0.0001; 0.3 mg: estimated difference: −7.71; confidence limits: −10.61 to −4.81; *P* < 0.0001). Also, Ketamine induced a significant reduction of 40 Hz ASSR power (Estimated difference: −4.32; confidence limits: −6.77 to −1.87; *P* = 0.0014). There were no significant differences with the comparison of vehicle at baseline and after the final washout neither in the MK‐801 group nor in the Ketamine group (*P* > 0.05) (Fig. 3F and 4D).

#### 40 Hz ASSR ITC

All three doses of MK‐801 reduced intertrial coherence (ITC) factor while the reduction was significant for the 0.1 mg and the 0.3 mg dose. (0.03 mg: estimated difference: −0.08; confidence limits: −0.14 to −0.01; *P* = 0.01; 0.1 mg: estimated difference: −0.15; confidence limits: −0.22 to −0.09; *P* < 0.0001; 0.3 mg: estimated difference: −0.18; confidence limits: −0.24 to −0.12; *P* < 0.0001). Ketamine 10 mg also significantly reduced the ITC factor (Estimated difference: −0.22; confidence limits: −0.26 to −0.18; *P* < 0.0001). Vehicle at baseline was not significant to vehicle after the final washout in the MK‐801 group (*P* > 0.05). However, there was a significant reduction of ITC in the Ketamine group, comparing vehicle at baseline and after the washout (Estimated difference: −0.08; confidence limits: −0.13 to −0.04; *P* = 0.0005 < 0.05; Fig. 3F and 4E).

#### Mismatch negativity

Only 50% of all 24 animals included in this study showed robust MMN at baseline conditions with a ratio of at least 0.5 for the standard/deviant ratio. To maintain a sufficient MMN for the evaluation of the NMDA receptor antagonist effects, we selected the 12 animals out of all 24 animals (MK‐801 group and Ketamine group) with a standard/deviant ratio of at least 0.5. So only for this readout, the selected animal group received both compounds ketamine and MK‐801 during the study. The selected group showed a significant pitch‐mediated MMN after vehicle injection (Fig. 3I 4F). Only 0.2 mg MK‐801 showed a significant reduction of MMN area under the curve compared to vehicle at baseline (Estimated difference: −478.40; confidence limits: −763.47 to −193.34; *P* = 0.0015). Ketamine 10 mg also induced a significant reduction in MMN (Estimated difference: −243.35; confidence limits: −386.76 to −99.94; *P* = 0.0027). This reduction of MMN was due to an amplitude reduction of the standard as well as the deviant potential. Vehicle at baseline was not significantly different to vehicle after final washout neither for the MK‐801 treatment nor for the ketamine treatment (*P* > 0.05) (Fig. 3G and 4F).

## Discussion

In this study, we validated in mice the leading biomarkers (auditory event‐related potentials (ERP), auditory event‐related oscillation (AERO), mismatch negativity (MMN) and auditory steady‐state response (ASSR)) relevant in schizophrenia research with a novel recording system which allows wireless recording of time‐locked EEG readouts in mice. In addition, this is to our knowledge the first preclinical study on ERP and AERO that uses commercially available software, frequently used in clinical research which in our opinion could help to increase transparency in terms of data analysis and potentially improve the reproducibility of preclinical ERP/AERO studies. We used epidural electrodes in this study since they are sensitive to neural activity from several distributed brain regions and are suggested to be more successful in recording true MMN in rodents (Harms et al. 2016). We used the NMDA receptor antagonist model with two different antagonists (Ketamine and MK‐801) to pharmacologically modulate the different biomarker, and we were able to demonstrate an impairment of all readouts after administration for both NMDA antagonists. To our knowledge, this is the first rodent study that used a wireless logger system to record event‐related EEG responses with a subsequent analysis that was performed entirely with commercially available software broadly used in the clinic.

### Auditory event‐related potentials

Patients with schizophrenia often report being unable to filter out distracting background noise/stimuli, leading to hypervigilance, impaired sensory processing/gating and problems in focusing attention (Javitt and Sweet 2015). There is evidence that these deficits might be linked to an impaired inhibitory feedback circuitry in the cortex and/or dysfunction of ascending thalamo‐cortical circuits (Freedman et al. 1983; Behrendt 2003; Cohen et al. 2015). Such deficits can be measured in humans as well as in animal models by measuring the neural response to auditory stimuli. The N1 deflection of an auditory ERP reflects the early phase of stimulus registration and processing [20]. The N1 deflection in an ERP is a response arising primarily from the auditory cortex (in humans 100 msec, in rodents 40 msec after presentation of an auditory stimulus). The N1 is sensitive to physical characteristics of the stimulus (e.g. duration, intensity, rise time) but less sensitive to contextual effects than later evoked potentials. Patients with schizophrenia have decreased N1 amplitude, which indicates impairment in early auditory sensory processing (Rissling and Light 2010). In the present study, we were able to confirm a significant reduction of the N1 amplitude in mice with both NMDA receptor antagonists as reported in previous preclinical studies (Maxwell et al. 2006; Saunders et al. 2012a; Nagy et al. 2016). However, we were not able to detect changes for the P1 and P3 amplitude as observed in other studies (Maxwell et al. 2006; Featherstone et al. 2012). These differences might be due to the fact that we used epidural electrodes in this study and others used mainly deep electrodes. We also investigated the gating index of the paired stimulus presentation. Auditory gating is impaired in schizophrenia patients (Freedman et al. 1983; Adler et al. 1986) but not affected by ketamine in healthy controls (van Berckel et al. 1998; Oranje et al. 2002; Hong et al. 2010). An interesting observation was the fact that the low dose of 0.03 mg MK‐801 increased the N1 amplitude, while the higher doses 0.1 mg and 0.3 mg reduced the N1 amplitude. Similar observations have been reported with the NMDA receptor antagonist Ketamine where low doses augment and high doses curtail the 40 Hz ASSR in the awake rat depending on the degree of NMDA channel blockade (Sivarao 2015). In the vehicle control group, there was a small but significant increase in the N1 amplitude over time which could reflect a habituation to the environment and a reduced stress level which could be responsible for a lower signal to noise ration reflected in the higher N1 amplitude. However, the effect of the NMDA receptor antagonists was robust enough to compensate this increase. Decreased gating can either result from a lack of suppression of the response to the second click or a decreased response to the first click. In patients with schizophrenia, impaired gating is more commonly observed due to a decreased response to the first tone. In this study, only relatively high doses of MK‐801 and Ketamine significantly disrupted the N1 gating function and increased the S2S1 peak ratio. This was mainly due to decreased amplitude evoked by the first click. These results are in accordance with previous studies that were unable to find an effect of lower doses of MK‐801 or Ketamine on sensory gating in rodents (Adler et al. 1986; de Bruin et al. 1999; Sullivan et al. 2015b) and let us suggest that due to the needed high doses, NMDA receptor antagonist‐induced sensory gating deficits are not the most suitable readout for translation into the clinic. However, impaired gating has been described in a mutant mouse model with reduced NMDA receptor function (Bickel et al. 2008) confirming at least a role of NMDA receptor function in sensory gating.

### Event‐related and basal gamma and theta oscillation

Neural oscillation and synchronization abnormalities have been suggested to play a role in the information and sensory processing deficits commonly seen in schizophrenia (Uhlhaas and Singer 2010; Gandal et al. 2012). The formation of functional networks through synchronized oscillation at the gamma band frequency is critically dependent upon the interplay between cortical pyramidal neurons and parvalbumin (PV)‐expressing interneurons which form the basis of neuronal excitatory and inhibitory networks (E/I‐balance) (Tatti et al. 2017). By modulating the level of responsiveness, these networks establish a transient links between ensembles of neurons (Fries 2015), the disturbances of which could underlie the pronounced cognitive and perceptual alterations in patients with schizophrenia (Uhlhaas and Singer 2015). In fact, alterations in evoked gamma response as well as in basal gamma oscillation are robust abnormalities in schizophrenia, and therefore such electrophysiological biomarkers of brain function hold great promise for schizophrenia as a tool to aid the diagnosis, prognosis, and translation from animal models to patients. In this study, we investigated the effect of two NMDA receptor antagonists on gamma oscillation during two different auditory stimulation paradigms (single‐click and auditory steady state). Our results demonstrate that both, ketamine and MK‐801 produce complex changes in the network oscillatory activity of neurons. These perturbations affect ongoing background activity as well as event‐related activities in opposite directions, reflected in an increased basal activity but reduced increase of task‐relevant evoked gamma activity. It is suggested that disinhibition of GABAergic interneurons that control the firing of pyramidal neurons, due to an acute blockade of NMDA receptors leading to hyperexcitation of glutamatergic neurons. (Moghaddam et al. 1997; Razoux et al. 2007). These observations have been reported in previous preclinical and clinical studies with NMDA receptor antagonists (Hong et al. 2010; Lazarewicz et al. 2010), so that it has been suggested that that the reduction in sensory‐evoked gamma activity produced by NMDA receptor antagonists is directly related to the excessive level of basal gamma activity (Kulikova et al. 2012). It is assumed that the increase in basal gamma oscillation by NMDA receptor antagonists represents electrophysiological “noise” which impairs the ability to detect and discriminate transient stimuli or task‐associated elevations in gamma oscillation (Kulikova et al. 2012; Hiyoshi et al. 2014). Consequently, the observed decrease of the evoked gamma power after an auditory stimulus is therefore suggested to further reduce the signal to noise ratio (Saunders et al. 2012b). We also observed a significant reduction of evoked theta oscillation which has been reported previously with Ketamine in rodents (Lazarewicz et al. 2010; Featherstone et al. 2012). Interestingly, we detected a reduced basal theta activity only with Ketamine but not with MK‐801, which might be due to specific pharmacological properties of these NMDA receptor antagonists. There is evidence that gamma oscillation is modulated by lower (e.g. theta) frequency bands (Lakatos et al. 2005; Canolty et al. 2006) and Schizophrenia patients have specific alterations in both gamma and theta oscillation(Kirihara et al. 2012). Since the hippocampus is the main generator of theta oscillation, a disruption of theta oscillation by NMDA receptor antagonists could lead to an increased background gamma activity (Gillies et al. 2002).

### Auditory steady‐state response (ASSR)

Networks of sensory cortical neurons can be entrained by a train of rhythmic auditory stimuli delivered at a particular frequency through the duration of stimulus presentation (Rager and Singer 1998; Oshurkova et al. 2008). The evoked oscillation is referred to as the auditory steady‐state response (ASSR) (Picton et al. 2003). In both human and animal models, the ASSR has been used to assess the functional integrity of neural circuits that support synchronization (Picton et al. 2003; Brenner et al. 2009; O'Donnell et al. 2013). An auditory stimulus is repetitively presented at a 40 Hz frequency and the cortical response to this stimulation is measured. We determined the power of the 40 Hz frequency band as well as the phase‐lock coherence. We were able to show a reduction of both endpoints after NMDA receptor antagonist application. NMDA receptor antagonists had no effect on lower frequency bands like 20 Hz (data not shown). It is not clear why there was a gradual decline of the ITC in the vehicle control group over the time of the study which results in a small but significant difference between baseline and washout. However, even though the washout did not completely reach baseline level, the compound effects are still very robust. The obtained results are in accordance with other preclinical studies showing a reduced ASSR after NMDA receptor antagonist intervention (Sivarao et al. 2013; Leishman et al. 2015). In contrast, some studies observed an increased 40 Hz ASSR phase‐locking after administration of MK‐801 and a reduction only at frequencies above 40 Hz (Vohs et al. 2012; Sullivan et al. 2015a). These discrepancies might be due to species differences or differences in the stimulation protocols. The reported dose‐dependent paradoxical effects of the NMDA receptor antagonist ketamine on ASSR where a low dose increased ASSR power and coherence and a high dose reduced both readouts (Sivarao 2015) were not reproduced with MK‐801 which might be due to higher receptor affinity of MK‐801 and resulting in a higher receptor occupancy with the low‐dose MK‐801. Since ASSR oscillations are time‐locked to stimulus and therefore highly reproducible when averaged across multiple trials, they are an excellent means to study the group response properties of underlying neurons. Human scalp‐recorded steady‐state oscillations are maximal at this frequency, suggesting natural resonance of the neural population (Galambos et al. 1981; Kwon et al. 1999). This idea is supported by blood oxygen‐dependent (BOLD) imaging studies that show that stimulation at 40 Hz evokes peak BOLD response, a measure synaptic activity (Pastor et al., 2002). ASSR measurements have become an important readout in clinical diagnostic and research, since consistent reductions are observed in ASSR power and phase‐lock coherence in schizophrenia. Preclinical studies suggest that the reductions in ASSR power may reflect reduced NMDAR activation within superficial pyramidal neurons and fast‐spiking interneurons (Carlen et al. 2012; Nakao and Nakazawa 2014). Only one study in humans used Ketamine and tested ASSR but detected an increased gamma power (Plourde et al. 1997). However, a temporally dynamic response was evident, potentially a reflection of the rapid pharmacokinetic properties of the drug (Sivarao 2015). Altered ASSR responses were also detected in preonset ultrahigh risk individuals and first episode Schizophrenia, therefore ASSR could also represent a biomarker for early detection and potentially prevention of psychosis (Tada et al. 2016) highlighting the potential of using noninvasively measured gamma‐band oscillations to provide insights into circuit abnormalities before psychosis onset.

### Mismatch negativity (MMN)

Deficits in MMN generation appear to be one of the most robust biomarkers in schizophrenia research and diagnostics (Todd et al. 2006, 2013; Light and Swerdlow 2015; Avissar et al. 2018; Javitt et al. 2018). Various studies have reported a reduced MMN in patients with schizophrenia utilizing different stimulation parameters e.g. pitch, duration, or intensity (Todd et al. 2008; Michie et al. 2016; Haigh et al. 2017). In contrast to most other readouts discussed in this study, MMN deficits appear to be relatively specific to schizophrenia. The MMN is an ERP component that reflects preattentive processing of successive auditory stimuli, in which a sequence of repetitive identical stimuli (standards) is interrupted by a physically different (deviant) stimulus. There is an ongoing debate about the existence of true deviance detection in rodents. However, by carefully applying criteria from human studies to distinguish between sensory‐specific adaptation (SSA) and true deviance detection in rodents there is strong evidence that the neurobiological mechanisms of MMN are translatable from humans to animals (Javitt 2015; Harms 2016; Lee et al. 2017, 2018; Featherstone et al. 2018; Harms et al. 2018). In the preclinical setting, a pitch deviance protocol is the mostly used paradigm. Such a protocol was also used in this study. For the analysis, we adapted a concept published for clinical application to ensure that individual variability in maximum appearance of MMN was considered (Beauchemin 2005). To have a sufficient window to show pharmacologically induced impairment of MMN, we defined robust MMN, if the ratio of standard/deviant was at least 0.5. Only 50% of all animals reached this criterion and about 30% of animals showed no MMN at all. In general, our data confirm that true MMN can be measured in mice as reported previously and we were able to show that MMN can be recorded with epidural electrodes and a wireless recording setup. However, the large interindividual variability in the magnitude of measured MMN makes its application as a preclinical biomarker very challenging. Large interindividual differences have also been reported in clinical studies (Beauchemin 2005). There are several clinical studies that reported reduced MMN amplitude after application of ketamine (Rosburg and Kreitschmann‐Andermahr 2016) as well as after application of MK‐801 in nonhuman primates (Javitt et al. 1996). This reduced MMN was mainly due to a reduction of the deviant response. Several preclinical studies reported a MMN disruption after acute NMDA receptor antagonist application; however, there is no consistency if this is a consequence of changed standard and/or deviant amplitude. Some studies report increased amplitude to the standard stimulus and a reduced amplitude to the deviant stimulus after NMDAR application (Ehrlichman et al. 2008; Tikhonravov et al. 2008); others reported only a change in the deviant response but not in the standard response (Lee et al. 2018), or the standard as well as the deviant amplitude reduced, but with larger reduction of the deviant amplitude (Harms 2016; Harms et al. 2018). There is also strong evidence that the key components of MMN, stimulus‐specific adaptation and deviance detection are differentiable in mouse cortical circuits and engage different types of interneurons (Hamm and Yuste 2016). In the present study, we observed a dose‐dependent decrease of the negative component for both, the deviant response as well as for the standard response which resulted in a reduced area under the curve MMN. However, with increasing doses of MK‐801 as well as with Ketamine, the evoked negative deflections of the standard as well as of the deviant became very small which consequently leads to a reduced MMN but does not exactly recapitulate the clinical picture of a specific deficit in deviance detection but merely a disruption of global signal processing, apparent also in the reduced ERP N1 amplitude described in this study. Therefore, we confirmed the existence of pitch‐mediated MMN in mice, but we experienced a limited robustness of MMN among individuals which represents a challenge by using this biomarker for standardized drug testing paradigms. In addition, due to the observed amplitude reduction of the standard as well as the deviant response after NMDA receptor antagonist application, the translational value as a model for reduced deviance detection in humans needs further investigation. Even though we used the flip‐flop sequence design for the stimulus presentations, a limitation of the current MMN study is that we did not conduct the many standards control paradigm or the cascade control sequence as recommended to control for stimulus‐specific adaptation versus true deviance detection (Harms et al. 2016; Featherstone et al. 2018) but we will perform these controls in future studies. We performed the deviant alone control sequence (Harms et al. 2016) but we refrained from using the data, since this approach does not take into account that the “adaptation load” is still larger for the deviant within an oddball sequence compared to the deviant alone condition (Taaseh et al. 2011) and does not appear to be a suitable control in human studies either (Fishman 2014).

## Conclusion

In summary, in this study we validated the Neurologger, a novel wireless recording device to measure a comprehensive selection of the most relevant clinical biomarkers in the context of auditory event‐related EEG. We investigated the acute effect of two different NMDA receptor antagonists in different doses on a wide variety of relevant time‐locked EEG readouts, like auditory event‐related potentials, basal and evoked gamma and theta oscillation, power and intertrial coherence of the 40 Hz auditory steady‐state response as well as mismatch negativity. We were able to show that both NMDA receptor antagonists induce significant impairment in most tested readouts, mimicking the clinical deficits observed in patients with Schizophrenia. In addition, this study confirms the suitability of the new wireless system to measure event‐related potentials with high precision, enabling analysis of all relevant time‐locked EEG readouts. We are convinced that the study can be of high translational value to bridge the existing gap between clinical and preclinical research since we analyzed all readouts with commercially available software with frequent application in clinical research and diagnosis. In addition, the performed analysis offers high transparency and reproducibility since all analysis steps have been performed with the commercially available software. Further, the new wireless logging system is a suitable device for recording of time‐locked EEG biomarkers in awake, freely moving mice and can be considered as a robust translational tool to investigate and validate novel therapeutic approaches in rodents regarding sensory processing deficits related to psychiatric disorders such as schizophrenia.

## Conflict of Interest

None declared.
